# Rotavirus and Norovirus infections among acute gastroenteritis children in Morocco

**DOI:** 10.1186/1471-2334-14-300

**Published:** 2014-06-03

**Authors:** Maria El Qazoui, Hicham Oumzil, Larbi Baassi, Nezha El Omari, Khalid Sadki, Saaid Amzazi, Mohamed Benhafid, Rajae El Aouad

**Affiliations:** 1 Immunology–Virology Department, National Institute of Hygiene, Ministry of Health, 27 Avenue Ibn Batouta, Rabat, Morocco; 2 Office of the Laboratories of the National Institute of Hygiene, Ministry of Health, 27 Avenue Ibn Batouta, Rabat, Morocco; 3 Laboratory of Biochemistry and Immunology, Department of Biology, Faculty of Sciences, 4 Avenue Ibn Batouta, B.P. 1014 RP, Rabat, Morocco; 4 UPR Immunology-School of Medicine and Pharmacy, Mohamed V University, Avenue Mohamed Belarbi El Alaoui Souissi, Rabat, Morocco

**Keywords:** Norovirus, Rotavirus, Acute gastroenteritis, Morocco

## Abstract

**Background:**

Acute gastroenteritis is a serious cause of child mortality and morbidity in resource-limited countries. A viral etiology is most common, and rotavirus and norovirus are reported to be the leading causative agents. There are still few epidemiological data on the simultaneous occurrence of these viruses in Morocco. The aim of this study was to provide useful epidemiological data on the gastroenteritis associated with rotavirus and norovirus among children aged less than 5 years.

**Methods:**

From January to December 2011, 335 samples were tested for rotavirus and norovirus using enzyme-linked immunosorbent assay, reverse-transcription-polymerase chain reaction (RT-multiplex PCR) and real-time RT-PCR. Partial sequences of the norovirus were phylogenetically analyzed to determine the genotype.

**Results:**

The overall rates of rotavirus and norovirus infections were 26.6% and 16.1%, respectively. Mixed viral infections were detected in 9 of 335 stool specimens (2.7%).

The most common genotype combination in the rotavirus strains was G1[P8] (51.7%), followed by G2[P4] (10.1%), G2[P8] (4.5%), G9[P8] (3.4%), G4[P8] (3.4%), and G1[P6] (2.3%). Among patients positive for norovirus, 42 (77.8%) tested positive for GII and 12 (22.2%) for GI. Thirty-three (78.6%) of the norovirus GII-positive cases were successfully characterized. Genotype GII.4 was the most prevalent (n = 27; 81.8%), followed by GII.3 (n = 2; 6.1%), GII.13 (n = 2; 6.1%), GII.16 (n = 1; 3%), and GII.17 (n = 1; 3%).

**Conclusion:**

This study suggests that in Morocco, norovirus is the most frequent cause of acute gastroenteritis after rotavirus, but further enteric viruses need to be integrated in the surveillance system so that a conclusion could be drawn.

## Background

Acute gastroenteritis is a very common disease in young children. It is characterized by massive inflammation of the gastrointestinal tract membranes, leading to frequent vomiting and/or diarrhea. Although regarded as a short-term intestinal disorder, acute gastroenteritis can cause severe dehydration, leading to further complications and hospitalization [1-3].

Enteric viruses are recognized as the most significant etiological agent of acute gastroenteritis, accounting for approximately 70% of episodes [2]. Four viral families are commonly associated with acute gastroenteritis: *Reoviridae* (group A rotaviruses), *Caliciviridae* (noroviruses), *Adenoviridae* (adenoviruses 40/41), and *Astroviridae* (astroviruses) [4-6]. Worldwide, rotavirus is considered the most common viral cause of severe acute gastroenteritis, causing 453,000 deaths and over 2 million hospitalizations among children under the age of 5 years [7].

Of the two major outer-shell proteins, VP7 defines the G genotype (G for Glycoprotein) and VP4 defines the P genotype (P for Protease-sensitive) and these are the main criteria upon which the dual classification system for the rotaviruses into P and G serotypes and genotypes [8]. Currently, 27 G genotypes (G1-G27) and 35 P genotypes (P[1]-P[35]) have been described for rotavirus [9]. To what extent each genotype defines an antigenically distinct VP7 or VP4 protein is not known.

Globally, the five most common G/P combinations among strains infecting humans are G1P[8], G2P[4], G3P[8], G4P[8], and G9P[8], [10]. Remarkably, the G/P types of the uncommon strains show wide variation from one region to the next. For instance, a surveillance program directed by the World Health Organization noted that in 2010 the predominant uncommon strains were G12P[8] and G12P[6] viruses in Southeast Asia; G2P[6], G3P[6], and G1P[6] viruses in sub-Saharan Africa; G1P[4] and G2P[8] viruses in the Western Pacific; and G9P[4] viruses in the Americas [11]. The uncommon strains that can be spread throughout the world to become common global strain are difficult to predict. Indeed, the G9P[8] strains represent the only clear example of a previously rare G/P genotype combination that has become dominant within the landscape of globally circulating rotavirus [12-14].

Norovirus is considered the second most frequent cause of severe childhood gastroenteritis after rotavirus [15]. Its prevalence in children with acute gastroenteritis is in the range of 6–48% [16]. Although more than 30 genotypes, within genogroups GI, GII, and GIV, can infect humans [17], a single genotype, GII.4, has been associated with the vast majority of norovirus-related outbreaks and sporadic cases of gastroenteritis worldwide [18-20]. The GII.4 norovirus strains undergo continuous processes of genetic/antigenic diversification and periodically generate novel strains through the accumulation of punctuate mutations or recombinations, and novel GII.4 variants emerge every 2–3 years [21,22].

Few data are available on the etiology of acute gastroenteritis among young children in Morocco. Only one study reports the detection of norovirus in shellfish in Morocco. Norovirus were detected in 30% of samples, with an equal representation of GI and GII strains, but were much more frequently found in cockles or clams than in oysters [23].

The report of a national survey performed in 1998, describing the causes of child deaths, revealed that a high rate of mortality among children aged less than 5 years was attributed to diarrheal diseases. The number of these deaths in 1 year is estimated to be 6000 [24]. In 2006, a national program for rotavirus surveillance was established by the Ministry of Health, in partnership with the World Health Organization, at four different sites. Data were collected during a 5-year survey and confirmed the rotaviral etiology of infective gastroenteritis among children aged less than 5 years in Morocco. Subsequently, isolated rotavirus strains have been investigated and their molecular epidemiology assessed [25]. These data supported the introduction of a rotavirus vaccine to the Moroccan National Immunization Program in 2010. The vaccine coverage rate was greater than 80% [26].

In countries where the rotaviral vaccine has been introduced, there has been a decline in morbidity related to rotavirus-associated gastroenteritis. However, a mathematical increase of the percentage involved in gastroenteritis cases related to norovirus has been reported. Norovirus has consequently been designated the predominant cause of gastroenteritis in hospitalized children [3,27,28].

To address the paucity of data regarding norovirus infections, we assessed the features of acute gastroenteritis related to rotavirus and norovirus in four regions of Morocco.

## Methods

### Setting

This prospective study was carried out as part of the regional rotavirus gastroenteritis surveillance network of the eastern Mediterranean region of the World Health Organization. The survey covered four geographically different regions of Morocco: the University Children’s Hospital in Rabat (central coastal region), Mohamed V Hospital in Tanger (northern coastal region), Al Farabi Hospital in Oujda (eastern region), and Prefectoral Hospital in Beni-Mellal (southern region).

### Specimen collection

In total, 335 fecal specimens were collected from children aged less than 5 years who were hospitalized or required medical care for acute gastroenteritis; Fourty six were from Rabat, 44 were from Tanger, 25 were from Beni-Mellal and 220 were from Oujda. The criteria for gastroenteritis were defined as follows: the acute onset of three or more loose stools, and/or two or more vomiting episodes a day that were not related to any other diagnosis, regardless of the fever conditions; stools with blood traces were excluded. A complete clinical examination was performed and a questionnaire was completed to collect the demographic data and clinical histories of the subjects.

### Viral investigation

Stools were collected within the first 48 h of admission. Rotaviral infections were screened with an enzyme-linked immunosorbent assay (ProSpecT Oxoid, Cambridgeshire, UK). The sensitivity of the test was 96.6% (83.0–99.9%) and its specificity was 96.4% (81.6–99.9%). Rotavirus-reactive samples were stored at −70°C until their molecular characterization with reverse transcription-polymerase chain reaction (RT-multiplex PCR) [24,25]. Rotaviral double-stranded RNA (dsRNA) was extracted from fecal suspensions using the QIAamp Viral RNA Mini Kit (Qiagen, Hilden, Germany), in accordance with the manufacturer’s instructions. The dsRNA samples were subjected to semi-nested multiplex RT-PCR using type-specific primers for VP7-G1, −G2, −G3, −G4, and -G9, and for VP4-P[4], −P[6], −P[8], and −P[10], as described previously [29,30]. All PCR products were examined by gel electrophoresis in 200 ml of 2% agarose gel containing 4 μg of ethidium bromide. Fragments of the VP7 gene (904 base pairs) or VP4 gene (876 base pairs) were amplified with the 9 con1-L/VP7-R primers [31] or con2/con3 primers, respectively [29].

Norovirus infections were assessed using real-time RT-PCR with oligonucleotide primers and probes as described previously [32,33], and tested for genogroups GI and GII. Viral RNA was extracted with the QIAamp Viral RNA Mini Kit (Qiagen). The norovirus-positive samples detected with real-time RT-PCR were also analyzed with conventional RT-PCR to allow sequence analysis. The primers sets G1SKF/G1SKR and G2SKF/G2SKR [34] were used to detect a fragment of the capsid gene (region C) of norovirus genogroups I and II, respectively. RT-PCR was performed with the OneStep RT–PCR kit (Qiagen), according to the manufacturer’s instructions.

The PCR products were sequenced with the same primers as were used for their amplification, with the ABI Prism BigDye Terminator Ready Reaction Cycle Sequencing Kit (Applied Biosystems Corporation, Foster City, CA, USA) and an ABI 3100 Genetic Analyzer (Applied Biosystems). DNA was sequenced in both directions using the BigDye Terminator cycling methodology (Applied Biosystems) and an ABI 3130xl Genetic Analyzer (Applied Biosystems). Sequences were aligned using ClustalW included in the Mega5.2 software [35]. A phylogenetic analysis was performed with 1000 bootstrapping replicates using the neighbor-joining method in the tree-builder tool of the same software.

### Nucleotide sequence accession numbers

The nucleotide sequence data were submitted to GenBank and assigned the accession numbers KJ162359–KJ162391.

### Ethical considerations

Informed consent was obtained from the parents or legal guardians of minors. The Ethics Office from the WHO and the Moroccan Ministry of Health deemed this surveillance as public health practice.

### Statistical analysis

Qualitative variables were compared with χ^2^ and Fisher’s tests (EpiInfo version 3.4) and the Mann–Whitney test was used to compare the Vesikari score values. The level of statistical significance was set at 95%. The Fisher’s test is particularly useful when the expected value is less than five.

## Results

### Study group

Among the 335 children enrolled in the study, 184 were boys and 151 were girls; the sex ratio (male/female) was 1.22. Their ages ranged between 1 and 59 months, with a median age of 12 months. The main clinical parameters related to acute gastroenteritis recorded in our study were the period of diarrhea, number of vomiting episodes, dehydration, and fever.

All the patients studied had diarrhea for periods of 1–15 days, with a median duration of 2 days. The median number of vomiting episodes was five episodes per day among all the children, with a range of 0–15 episodes per day. Sixty-four percent of sufferers showed a high rate of vomiting episodes, and 61.3% experienced fever. Of the children enrolled, 30% showed moderate dehydration and 40% severe dehydration. Children aged less than 12 months were the most severely affected, with an estimated dehydration rate of 49%.

### Rotavirus infections

**Figure 1:**
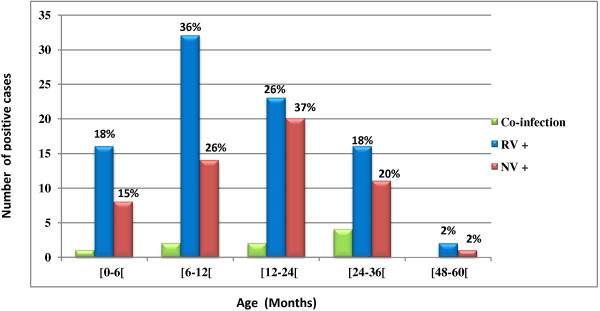
Age distribution of rotavirus and norovirus infected cases.

The estimated rate of the rotaviral infection was 26.6% (89/335). The age of the children affected with rotavirus was between 2 and 59 months, with a median age of 12 months. The great majority (36%) of rotaviral infections occurred in children aged 6–12 months (Figure 1). Rotavirus was detected at a higher rate in male stool samples (53.9%) than in female samples (46.1%). The sex ratio (male/female) was 1.17. However, there was no significant correlation between sex and rotaviral infection. The median duration of diarrhea experienced by children with rotaviral infections was 6 days, and vomiting episodes occurred at a median rate of six events per day.

**Figure 2:**
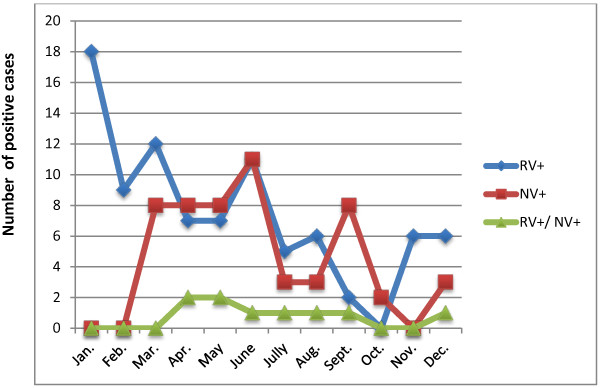
The seasonal distribution of rotavirus and norovirus infections between January and December 2011.

Severe dehydration was identified in 44 of 78 patients infected with rotavirus (56.41%). Twenty-six patients (33.33%) experienced moderate dehydration, but 8 of the 78 rotavirus-infected children (10.26%) showed no symptoms of dehydration (Table 1).Although rotavirus infections were recorded throughout the year, the number of cases tended to peak in January (Figure 2).

**Table 1:** Clinical features of rotavirus and norovirus infection

	Rotavirus (N = 78)*	Norovirus (N = 43)**	Negative (N = 192)***	
**Maximum number stools per day**				
1-3	01 (01.28%)	03 (06.98%)	16 (08.33%)	
4-5	21 (26.92%)	12 (27.91%)	63 (32.81%)	
≥ 6	56 (71.79%)	28 (65.12%)	113 (58.85%)	
**Diarrhea duration (days)**				
1-4	74 (94.87%)	40 (93.02%)	168 (87.50%)	
5	03 (03.85%)	00 (00.00%)	14 (07.29%)	
≥ 6	01 (01.28%)	43 (06.98%)	10 (05.21%)	
**Number vomiting episodes per day**				
0	05 (06.41%)	05 (11.63%)	50 (26.04%)	
1	00 (00.00%)	03 (06.98%)	07 (03.65%)	
2-4	16 (20.51%)	09 (20.93%)	50 (26.04%)	
≥ 5	57 (73.08%)	26 (60.47%)	85 (44.27%)	
**Vomiting duration (days)**				
0	05 (06.41%)	05 (11.63%)	50 (26.04%)	
1	19 (24.36%)	07 (16.28%)	31 (16.15%)	
2	26 (33.33%)	14 (32.56%)	57 (29.69%)	
≥ 3	33 (42.31%)	17 (39.53%)	54 (28.13%)	
**Temperature (°C)**				
37	25 (32.05%)	17 (39.53%)	79 (41.15%)	
37.1-38.4	05 (06.41%)	02 (04.65%)	15 (07.81%)	
38.5-38.9	02 (02.56%)	11 (25.58%)	34 (17.71%)	
≥ 39.0	46 (58.97%)	13 (30.23%)	64 (33.33%)	
**Dehydration**				
Mild	08 (10.26%)	17 (39.53%)	67 (34.90%)	
Moderate	26 (33.33%)	13 (30.23%)	55 (28.65%)	
Severe	44 (56.41%)	13 (30.23%)	70 (36.46%)	
**Treatment**				
Rehydration	07 (08.97%)	12 (27.91%)	45 (23.44%)	
Hospitalization	71 (91.03%)	31 (72.09%)	147 (76.56%)	
				**P value**
**Vesikari score**	14.74		12.38	< 0.0001
		13.12	12.38	0.26

*89 rotavirus positives samples-(9 co-infections + 2 data missing) = 78.

**54 norovirus positives samples-(9 co-infections + 2 data missing) = 43.

***201 negatives samples-(9 data missing) = 192.

### Norovirus infections

The estimated rate of noroviral infection was 16.1% (54/335). Rabat and Tanger which are coastal cities had the highest percentages of norovirus positivity, during the study period. The difference between the coastal sites and non-coastal sites was statistically significant (Table 2).The age of the children affected was between 2 and 48 months, with a median age of 14 months. The highest rate was noted in children aged 12–24 months (37%) (Figure 1). Sample stools from male children showed a higher rate of norovirus infection (57.4%) than those from female children (42.6%), but the difference was not statistically significant. The sex ratio (male/female) of norovirus patients was 1.35.

**Table 2:** Distribution of norovirus positive cases in the 4 sites of the study, in 1 year survey

**Provinces**	**Localization area**	**Gastroenteritis samples**		**Number of positive cases**		**Positivity (%)**	**P value**
**Rabat**	Coastal cities	46		13		28.3	
**Tanger**		44	90	11	24	25	
**Beni-Mellal**	Non-coastal cities	25		1		4	0.001
**Oujda**		220	245	29	30	13.2	
**Total**		335		54			

The median period of diarrhea among the norovirus-infected patients was 2 days and the median number of vomiting episodes was five events per day. Of the 43 detected cases of noroviral infection, severe dehydration occurred in 13 (30.23%) children and 13 (30.23%) showed moderate dehydration. No dehydration was recorded in 17 norovirus-infected children (39.53%) (Table 1). Seasonal follow-up of the norovirus-infected patients showed a peak incidence in June (Figure 2).

### Dual norovirus and rotavirus infections

Dual norovirus and rotavirus infections were confirmed in 9 of 335 (2.7%) patients and were recorded in children with a median age of 24 months. The median period of diarrhea was 3 days and the median number of vomiting episodes was four per day.Of the nine patients with mixed rotavirus–norovirus infections, two showed severe dehydration (22.22%) and four (44.44%) showed moderate dehydration. Three of the nine coinfected children (33.33%) showed no signs of dehydration. The majority of rotavirus-norovirus dual infections were recorded between April and May (Figure 2).

### Rotaviral strain characterization

**Figure 3:**
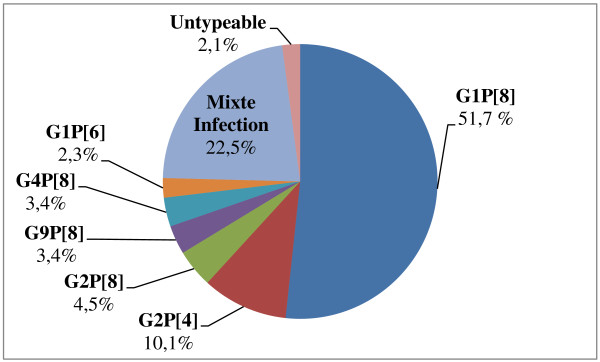
Distribution of G/P genotypes combinations in Morocco, from January to December 2011.

The rotaviral strains isolated from stool samples were subjected to molecular characterization, and P and G types were assigned to 89 isolates with RT-PCR. The most common genotype combination in the rotaviral strains was G1P[8] (51.7%), followed by G2P[4] (10.1%), G2P[8] (4.5%), G9P[8] (3.4%), G4P[8](3.4%) and G1P[6] (2.3%). A high prevalence of mixed strains was found (22.5%) and 2.1% of strains could not be typed (Figure 3).

### Noroviral strain characterization and phylogenetic analysis

Analysis of the genetic diversity of the noroviral strains in our study showed extensive cocirculation of various genotypes between January and December 2011. Among the children positive for norovirus, 42 (77.8%) carried genotype GII and 12 (22.2%) carried genotype GI. A subset of the norovirus samples positive for GII was selected for sequence analysis, and 33 were successfully characterized in region C of the viral genome. The prevailing genotype was GII.4, accounting for 81.8% of infections (n = 27), whereas genotypes GII.3 and GII.13 each accounted for 6.1% of infections (n = 2).

**Figure 4:**
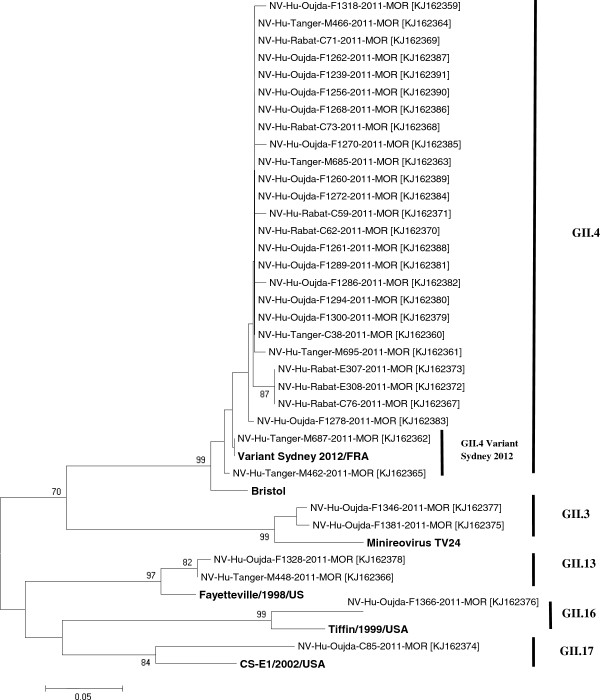
**Phylogenic analysis of norovirus sequences.** The genetic relationship is based on a 322-nucleotides fragment of Region C (VP1). The tree was generated using the neighbor-joining method and the bootstrap values from 1000 replicates were shown on each branch. The sequence accession numbers are given in parentheses. The reference strains were from NCBI Genbank: Bristol(X76716), Fayetteville (AY113106), MinireovirusTV24 (U02030), CS-E1/2002/USA (AY502009), Tiffin-1999 (AY502010) and Variant Sydney 2012/FRA (KF008239).

Other genotypes, GII.16 and GII.17, were each represented by one strain (n = 1, 3%). The nucleotide sequence analysis showed that GII.4 was the most prevalent genotype. The variant strain designated “GII.4 variant Sydney 2012” was identified in two samples. A sequence analysis of these two samples based on a 302-bp sequence of the capsid gene (C-region) showed 99% identity with the variant Sydney 2012.A representative phylogenetic tree based on the nucleotide sequences of the capsid region (C-region) was constructed with the neighbor-joining method (Figure 4).

## Discussion

In this study, acute gastroenteritis with a rotaviral or noroviral etiology was investigated in children aged less than 5 years. This is the first study to investigate both viruses simultaneously in Morocco. Of the 335 children with acute gastroenteritis enrolled in the study, 26.6% (n = 89) tested positive for rotavirus and 16.1% (n = 54) for norovirus. Our results are consistent with several studies undertaken elsewhere, indicating that norovirus is the most common cause of acute gastroenteritis, after rotavirus [36-38].

After the introduction of a rotavirus vaccine in the United States, hospitalizations related to acute gastroenteritis were 45% lower than 2 years earlier [39]. A substantial decline in rotavirus-associated acute gastroenteritis was also observed. Therefore, norovirus is now considered the leading cause of severe gastroenteritis outbreaks in the United States [27,40].

In Morocco, prior the implementation of the national immunization program with rotavirus vaccine, circulating isolates of rotavirus were genotyped in our facilities. From June 2006 to May 2009, 1388 stool samples were collected from children less than 5 years of age admitted for diarrhea in different regions of Morocco. Overall, 41.7% (579 of 1388) of the stools samples tested were positive for rotavirus. Genotyping of 548 (95%) samples demonstrated that G1P[8] (55%) was the most prevalent strain, followed by G9P[8] (11.3%), G2P[4] (9.1%), G4P[8] (0.9%), and G3P[8] (0.4%). Several other strains were identified including G1P[4] (0.2%), G1P[6] (0.9%), G2P[6] (4.3%), G2P[8] (0.2%), G3P[6] (0.4%), G3P[4] (0.2%), and G9P[6] (0.2%). A high prevalence of mixed infections was found (15% of all samples) of which G1G2P[8] (4%) and G1G3P[8] (3.6%) accounted for the majority. Considerable diversity of rotavirus genotypes was present among circulating strains in Morocco [25]. After introduction of rotavirus vaccination in Morocco, the most common genotype combination of rotavirus strains was G1[P8] (51.7%), followed by G2[P4] (10.1%), G2[P8] (4.5%), G9[P8] (3.4%), G4[P8] (3.4%) and G1[P6] (2.3%). A high prevalence of mixed strains (22.5% of the total) was found. The prevalence of rotavirus gastroenteritis decreased from 41.7% to 26.6%. The involvement of the vaccine introduction in the decreasing of the prevalence and the changing in molecular diversity of rotavirus strains in Morocco needs further study. The selective pressures coming from the vaccine may be subtle; many years are required before making a conclusion concerning this phenomenon [41].

Because the Moroccan childhood immunization program was implemented with a rotavirus vaccine in 2010, it is likely that norovirus-associated gastroenteritis will increase relative to rotavirus-associated gastroenteritis, as described by Patel et al. [15] and Payne et al. [27]. A prospective survey is required to determine the point at which the switch in this trend occurs.

Both viruses were detected in all age groups examined in this study. The peak detection rate occurred between 6 and 12 months of age for rotavirus, with a median age of 12 months, and between 12 and 24 months of age for norovirus, with a median age of 14 months, as was also shown in a French study [36]. These findings confirm that both viruses usually occur in early childhood, which may indicate that protective immunity is present after the age of 2 years [42], and also highlights the importance of implementing prevention strategies in the early years of life. The overall severity score was significantly higher for rotavirus gastroenteritis than for norovirus infections. Similar results have been reported in Spain, Poland, and Libya [3,37,43]. Those studies showed that rotavirus-infected children experienced longer periods of diarrhea and were more severely dehydrated than children infected with norovirus.

During this 1-year survey, cases of rotavirus- and norovirus-associated gastroenteritis were recorded throughout the year, except in October and February, when no case of rotavirus and norovirus was detected, respectively. We observed one peak for rotavirus in January and two peaks for norovirus, in June. The increased rates of noroviral infection in spring and summer have been documented previously in Libya and Spain [43,44]. The summertime peak may be explained by the emergence of a new virus variant, as reported by Lopman et al. [45].

In Morocco, spring and summer seasons are commonly associated with increased consumption of seafood particularly oysters, which is known as a risk factor for acquiring norovirus infection [23,46]. Rabat and Tanger which are coastal cities had the highest percentages of norovirus positivity, during the study period. The study needs to be longer so that a conclusion could be drawn.

Norovirus GII is predominantly responsible for gastroenteritis worldwide, as described in most studies [47-52], and our findings (77.8% GII and 22.2% GI) are consistent with these findings. A subset of norovirus-positive GII samples (n = 33) was sequenced and grouped into five different genotypes: GII.4 (*n* = 27) was the predominant genotype, followed by GII.3 (*n* = 2) and GII.13 (*n* = 2), GII.16 (*n* = 1), and GII.17 (*n* = 1). Several global studies have demonstrated the prevalence of the GII.4 genotype and our results are consistent with them [18,19,43,53].

Unexpectedly, our results showed the presence of the GII.4 variant Sydney2012 in two samples, from the northern region of Morocco (GenBank accession numbers KJ162365 [Tanger/M462/2011/MOR] and KJ162362 [Tanger/M687/2011/MOR]). This variant was first identified in March 2012 in Australia [54] and in December 2012 in Denmark [55], one year after the sampling for our study. This supports the idea that patients with sporadic gastroenteritis are a reservoir for emerging epidemic noroviral strains [52].

Dual rotavirus-norovirus infections were detected in 2.7% of our study samples. Approximately the same rate was reported (2.1%) in a Polish study [3], but our rate is lower than that reported in France by Tran et al. [36]. As reported in the present study and the French study [36], there was no significant correlation between the severity of gastroenteritis and combined infections.

## Conclusion

Our findings highlight the need to strengthen the surveillance of gastroenteritis and extend it to others regions in Morocco. This will allow us to monitor changes in the epidemiology of rotavirus- and norovirus-associated gastroenteritis and to identify dynamic shifts in the circulating strains.

## Competing interests

The authors declare that they have no competing interests.

## Authors’ contributions

ME, MB, SA, and RE designed the epidemiological study, participated in its design and coordination, and gave their final approval to the version to be published. ME performed the molecular studies, participated in the sequence alignment, and drafted and edited the manuscript. HO and KS were involved in drafting the manuscript. NE performed the laboratory tests on the human stool samples. LB performed the statistical analysis and participated in its design. All authors read and approved the final manuscript.

## Pre-publication history

The pre-publication history for this paper can be accessed here:

http://www.biomedcentral.com/1471-2334/14/300/prepub
